# Epigenetic study of the long-term effects of gulf War illness

**DOI:** 10.3389/fgene.2025.1553410

**Published:** 2025-06-18

**Authors:** B. C. Jones, J. P. O’Callaghan, D. G. Ashbrook, L. Lu, P. Prins, W. Zhao, K. Mozhui

**Affiliations:** ^1^ Department of Genetics, Genomics, and Informatics, University of Tennessee Health Science Center, Memphis, TN, United States; ^2^ Centers for Disease Control and Prevention, National Institute for Occupational Safety and Health, Morgantown, WV, United States; ^3^ Department of Preventive Medicine, University of Tennessee Health Science Center, Memphis, TN, United States

**Keywords:** MBD-seq, Forward genetcs, QTL (loci of quantitative traits), BXD mice, neuroinflammation

## Abstract

**Introduction:**

Gulf War Illness is a chronic multisymptomatic disorder that affects as many as 25-35% of the military personnel who were sent to the Persian Gulf war in 1991. The illness has many debilitating symptoms, including cognitive problems, gastrointestinal symptoms, and musculoskeletal pain. Those so afflicted have been sick for more than 30 years and, therefore, it has become imperative to understand the etiology of Gulf War Illness and then produce treatments to ease the symptoms. We hypothesized that the length of the disease was reflected in epigenetic modification of possibly several genes related to the symptoms.

**Methods:**

We subjected male and female mice from 11 BXD strains to combined corticosterone and the sarin surrogate, diisopropylfluorophosphate, to emulate the physiological stress of war and the potential exposures to organophosphate pesticides and nerve agent in theater. Three hundred days after treatment, we used Methyl-CpG-binding domain sequencing (MBD-seq) to assay genome-wide methylation.

**Results:**

The analysis revealed 20 methylated genes, notably Eif2b5, that regulates myelin production.

**Discussion:**

Loss of myelin with accompanying musculoskeletal pain is a major symptom of Gulf War Illness. Our work demonstrates multiple genes were methylated by exposure to organophosphates and glucocorticoids. These genes point to biochemical mechanisms that may be targets for therapeutic intervention.

## Introduction

In 1991, a 42-nation coalition led by the United States, initiated combat against Iraq because of its invasion of Kuwait (Engelhardt, 2019). Nearly one million personnel were involved and of those who participated in combat actions, between 25 and 35 percent became sick with a multi-symptom malaise now called Gulf War illness (GWI). Symptoms include gastrointestinal, respiratory, fatigue and cognitive problems (Fappiano and Baraniuk, 2020). One of the hypotheses concerning the cause of the syndrome is exposure to organophosphates (OP), including sarin and chlorpyrifos, combined with increased circulating glucocorticoids as might be expected in the stress of combat. Moreover, for many of the afflicted veterans, the symptoms have persisted for more than 30 years since the conflict ended (Petry et al., 2024). Earlier research from our group presented evidence for genetic contributions to individual differences in susceptibility to the acute effects of exposure (Jones et al., 2020; Xu et al., 2020; Gao et al., 2020). In that series of experiments, we identified two candidate genes underlying individual differences in susceptibility to the exposures experienced by the troops in theater. That many of the afflicted veterans experience symptoms more than 30 years after cessation of the conflict poses a different, but important feature of GWI. The question is what is the reason these individuals are sick for so long? One highly likely possibility is the exposure modified the expression potential of one or more genes. Accordingly, we performed small studies to examine the possible methylation of genes, thus reducing their expression potential. The first of these involved the acute effects of a sarin surrogate coupled with a glucocorticoid (Ashbrook et al., 2018) in a single inbred mouse strain, C57BL/6J, one of the foundation strains for the genetic reference family of recombinant inbred mouse strains, the BXD group (Peirce et al., 2004). The second study involved the two foundation strains for the BXD group, C57BL/6J and DBA/2J (Mozhui et al., 2023). The basis for the present study was the observation in two previous studies that one of the targets for GWI exposure profile in our animal model was myelin (Ashbrook et al., 2018; Xu et al., 2020). Importantly, one of the pathophysiological chronic signs in afflicted GWI veterans is diminished myelin with associated muscular pain (Heaton et al., 2007; Chao et al., 2015; Belgrad et al., 2019). Accordingly, the major hypothesis that we proposed is that chronic GWI has major impact on myelination and the genes that regulate oligodendrocytes. In this study, we performed the same treatments in our genetic reference population of mice, exposure to corticosterone followed by treatment with a sarin surrogate but harvesting the hippocampus for analysis of genome-wide methylation 43 weeks after the sarin surrogate treatment, to model the years-long persistent nature of the disorder in ill veterans. Here we report the results from a study that expanded the number of strains and time after treatment.

## Methods

### Mice and treatment

Male and female mice from11 BXD strains age 60–65 days of age were the subjects for this study. All subjects received corticosterone in their drinking water (20 mg% w/v) for 7 days. On the eighth day, the animals received 4.0 mg kg^−1^ i.p. diisopropylfluorophosphate (DFP), a sarin surrogate. These are the same doses of corticosterone and DFP reported in our previous work on the acute effects of these treatments (Jones et al., 2020) and developed by O’Callaghan and colleagues (O'Callaghan et al., 2015). Over the next 43 weeks and for the first 12 weeks on alternate weeks, the animals received corticosterone as above in their drinking water. On the 42nd week, the animals received corticosterone in their drinking water. These repeated corticosterone treatments were done to prime and maintain the neuroinflammatory effect of the original corticosterone/DFP treatment. Glucocorticoids are regarded as having anti-inflammatory effects; however, they can also show pro-inflammatory effects, (Frank et al., 2010; Bolshakov et al., 2021). One week following the last corticosterone treatment, the mice were euthanized, and the hippocampus harvested. The DNA was extracted from the tissues and prepared for Methyl-CpG-binding domain sequencing (MBD-seq) to identify genome-wide methylation and RNA extracted for analysis. The interval between DFP treatment and tissue harvest was based on mouse-human age comparison to evaluate mice at an equivalent human age in the early 50s based on Jackson Laboratory website.

### Design summary

Week 1, days 1–7, Corticosterone in the drinking water.
Week 2, day 1 (8 days since corticosterone treatment) i.p. injection of 4 mg/kg DFP.
Week 3, days 2–7 no corticosterone in the drinking water.
Weeks 4–12 Corticosterone in drinking water on alternate weeks beginning with week 4
Weeks 13–41 No treatments.
Week 42 Corticosterone in drinking water.
Week 43 days 1, euthanize mice, harvest tissue.
Mouse n = 5 per strain, sex, and treatment.

### Tissue harvest and sample preparation

Genomic DNA was extracted from the hippocampus using the Quick-DNA/RNA Miniprep Plus kit (Zymo Research, Irvine, CA, United States) and checked for purity and quantity using a NanoDrop spectrophotometer (ThermoFisher Scientific, Waltham, MA, United States), and a Qubit™ fluorometer and the dsDNA BR (Broad Range) Assay kit (Invitrogen). Affinity based CpG enrichment was done using the Invitrogen MethylMiner Methylated DNA Enrichment Kit (ThermoFisher Scientific, Waltham, MA, United States), which relies on the methyl-CpG binding domain protein 2 (MBD2) protein to capture DNA fragments containing methyl-CpGs. MBD2 preferentially binds to methylated CpGs, and this depletes the DNA sample of DNA regions without CpGs, and enriches for methyl-CpGs (Aberg et al., 2018; Aberg et al., 2020) First, 1 µg of DNA in 110 μL low TE (tris-EDTA) buffer was sheared to∼150 bp fragments using a Covaris S2 ultrasonicator (Covaris, Woburn, MA, United States). Sonication settings were the same as described by Sandoval-Sierra (Sandoval-Sierra et al., 2020) with cycle/burst of 1 for 10 cycles of 60 s, duty cycle of 10%, and intensity of 5.0. DNA fragment size and quality were assessed using the Agilent Bioanalyzer 2100 (Agilent, Santa Clara, CA, United States). MBD-capture reaction was done according to the standard manufacturer’s protocol, followed by a single step elution with 2 M NaCl solution. The enriched DNA was then reconcentrated by ethanol precipitation, and the final concentration of methylated-CpG enriched DNA ranged from 0.17 to 2.1 ηg per μl (0.87 ± 0.39).

### Sequencing and initial data processing

Sequencing was performed to 40 million reads per sample (150 paired-end) on Illumina NovaSeq 6000 (Illumina, San Diego, CA, United States). *Mus musculus* (mouse) reference genome (GRCm38) and gene model annotation files were downloaded from the Ensembl genome browser (https://useast.ensembl.org/). Indices of the reference genome were built using STAR v2.5.0a (Dobin et al., 2013) and paired-end clean reads aligned to the reference genome.

Following alignment, the bam files were loaded to the MEDIPS R package (version 1.52.0) (Lienhard et al., 2014). The parameters for the MEDIPS.createSet function were: uniq = 1, extend = 150, window_size = 150, shift = 0, and the genome used was BSgenome.Mmusculus.UCSC.mm10. DNA methylation quantification was based on number of read counts within a 150 bp non-overlapping bins. The local CpG density (the coupling factor or CF) was computed using the MEDIPS.couplingVector function, and the read counts were normalized to the CF using the function MEDIPS.meth. We excluded all bins with CF = 0 (i.e., no CpG in the 150 bp bin), and this resulted in 10,647,424 bins. The raw read counts were the further processed using the EdgeR R package (version 3.42.4) (Robinson et al., 2010). Prior to statistical tests, we further filtered out bins with counts per million (CPM) less than 1 per library. This retained 432,694 DNA methlation regions that were normalized by the library size using the normLibSizes function. These 150 bp bin read counts were converted to logRPKM values. We then used the ChIPseeker (Yu et al., 2015) and AnnotationDbi (ref: see below for the website) R packages to annotate the bins for relevant gene information and genomic features (e.g., promoters, UTRs, introns, intergenic, etc.) (Pagès et al., 2024).

### Statistical analyses

We performed multivariate regression for an epigenome-wide association study (EWAS). The primary goal was to identify the main effect of treatment in a genetically complex population. For initial quality check, we applied a principal component analysis (PCA), and the top PCs were used to detect possible outliers, and verify strain identify and sex. Background strain has a strong effect on DNA methylation and these are captured by the top PCs, and this gives us a variable to adjust for background genotype during EWAS for the main effect of treatment (Sandoval-Sierra et al., 2020). For the initial EWAS, we used the following regression model: glm(y_i_ ∼ treatment + sex + body weight + PC1 + PC2 + PC3), where y_i_ is methylation level at CpG region 1–432,694. Following the initial identification of CpGs associated with the main effect of treatment, we then examine the strain differences as a *post hoc* analysis.

## Results

### Effect of CORT + DFP on DNA methylation

**FIGURE 1:**
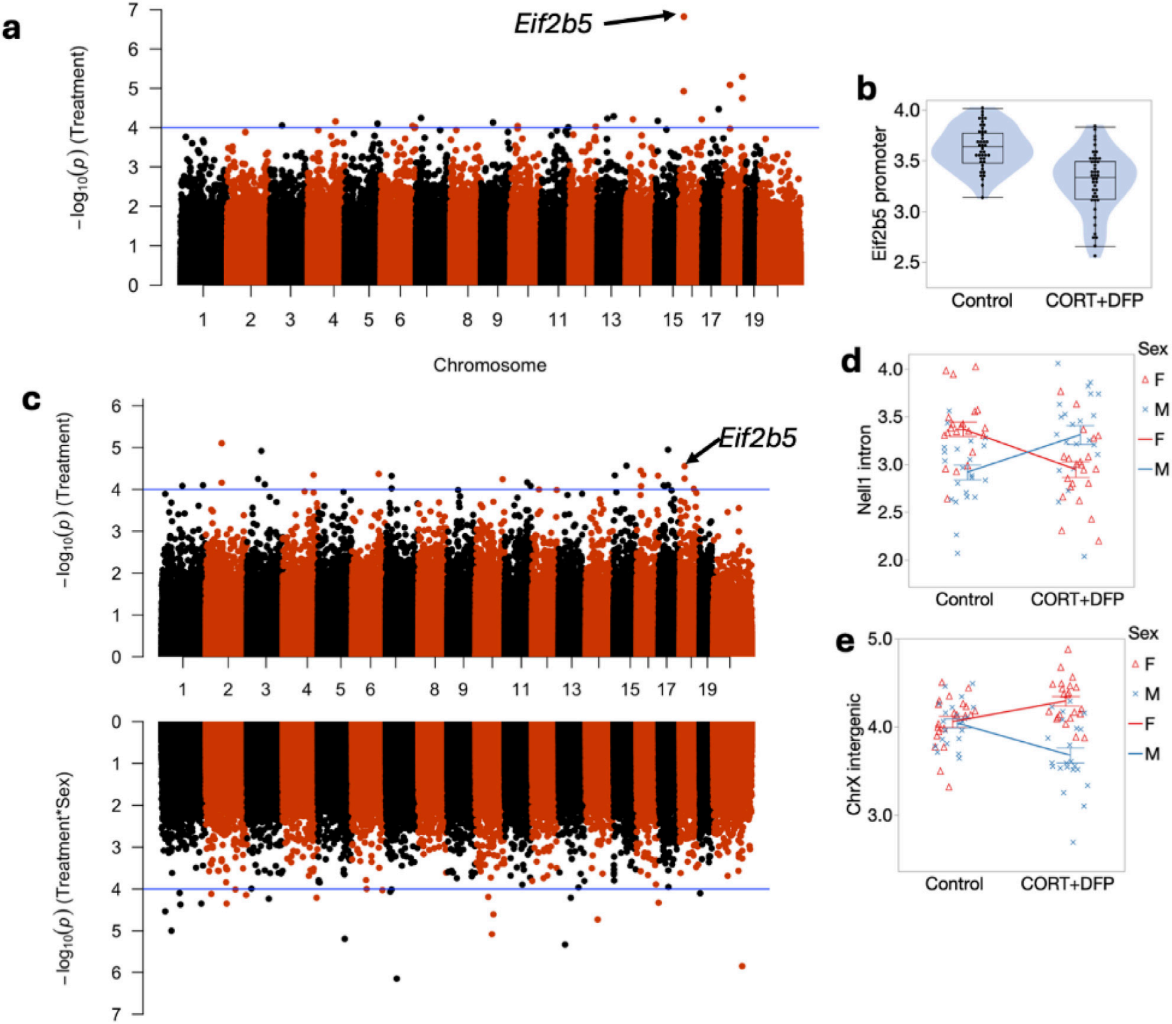
Manhattan plot of differentially methylated CpG regions. **(a)** main effect of CORT + DFP; **(b)** Effect of CORT + DFP on expression of the promoter region of *Eif2b5*; **(c)** Manhattan plot following addition of sex X treatment; **(d)** sex differences in methylation of *Eif2b5*; **(e)** sex differences in treatment in an intergenic region on X chromosome.

To define site specific changes induced by the long-term exposure to CORT + DFP, we performed an epigenome-wide association study (EWAS) to compare between the treatment and control groups. For the first test (EWAS 1), we examined the main effect of treatment, with sex and body weight as cofactor, and adjusted for the top 3 PCs as proxies of genetic heterogeneity and other sources of variance. At a nominal unadjusted p = 0.0001, 34 CpG regions were altered in methylation by treatment (Data S1). The strongest main effect of treatment was on the promoter methylation of the *Eif2b5* gene (Figure 1a). This region showed no difference between the sexes and was significantly decreased in methylation by CORT + DFP (Figure 1b). Analysis of variance showed a significant treatment effect (F1,65 = 25.62, p < .001). The main effect for strain and strain X treatment interaction were not significant (F10,65 = 1.55, p < .2; F10,65 = 1.02, p < .4, respectively). The top 20 CpG regions associated with the CORT + DFP treatment are presented in Table 1. Many of the CpG regions were in intergenic sites, but also included CpGs located in the introns or exons of *Ctif*, *Cdh6*, etc. We followed up the main EWAS with a regression that include the sex-by-treatment interaction (EWAS 2). The addition of this additional parameter reduced the associations for the main effect of treatment (Figure 1c). However, as a sensitivity test, the association with treatment for most of the CpG regions shown in Table 1 remained consistent at p < 0.001. This also revealed CpGs that had evidence of sex-by-treatment interactions. The strongest interaction effect was for a CpG region in the intron of *Nell1* (alternatively, a promoter of the *Nell1os*). While this region shows no difference by treatment when all samples are pooled, but when stratified by sex, females show a reduction in methylation, while males show an increase in methylation with treatment (Figure 1d). An intergenic region on chromosome X showed a contrasting effect on treatment between the two sexes (Figure 1e). The top 20 CpG regions with treatment-sex interaction are in Table 1. None of these were associated with treatment based on EWAS 1.

**TABLE 1:** Methylated genes identified for Main effect of CORT + DFP treatment and sex by treatment interaction.

				EWAS 1^a^ Treatment effect		EWAS 2^a^ Treatment effect	
GeneSymbol	Bin	Bin Bp	Annotation	coef	p	coef	p
*Eif2b5*	16	20501551	Promoter (2–3 kb)	0.33	1.5E-07	0.35	3.5E-05
*Ctif*	18	75624151	Exon (exon 3 of 12)	−0.35	5.0E-06	−0.34	1.3E-03
*2700062C07Rik*	18	24484351	Distal Intergenic	0.30	8.2E-06	0.39	2.8E-05
*Or2aj6*	16	19650151	Distal Intergenic	0.30	1.2E-05	0.36	1.4E-04
*Ctif*	18	75624001	Exon (exon 2 of 2)	−0.33	1.8E-05	−0.21	3.8E-02
*2410021H03Rik*	17	69325201	Intron (intron 2 of 2)	−0.21	3.4E-05	−0.16	2.2E-02
*Nsun2*	13	69283501	Distal Intergenic	0.25	5.1E-05	0.19	1.9E-02
*Nlrp4e*	7	23315701	Intron (intron 1 of 8)	−0.26	5.7E-05	−0.33	2.7E-04
*Gfod1*	13	43327351	Distal Intergenic	−0.29	5.9E-05	−0.13	1.6E-01
*Hlcs*	16	94243501	Intron (intron 1 of 6)	0.29	6.2E-05	0.20	3.4E-02
*Dph3*	14	32112151	Distal Intergenic	0.29	6.2E-05	0.31	2.2E-03
*Cdh6*	15	13034251	Exon (exon 12 of 12)	0.27	6.8E-05	0.33	3.2E-04
*Armh1*	4	117353251	Intron (intron 2 of 14)	0.25	7.0E-05	0.27	2.2E-03
*Zc3h12c*	9	52416301	Exon (exon 2 of 2)	0.27	7.5E-05	0.35	2.1E-04
*Stag3*	5	138321601	Distal Intergenic	−0.30	8.0E-05	−0.33	1.8E-03
*Maml3*	3	51988651	Exon (exon 2 of 2)	−0.23	8.8E-05	−0.33	5.6E-05
*Lrp6*	6	134587651	Distal Intergenic	0.31	9.1E-05	0.34	1.9E-03
*Sult3a2*	10	33747001	Distal Intergenic	0.23	9.1E-05	0.30	2.6E-04
*Kif26a*	12	112277851	Distal Intergenic	0.28	9.4E-05	0.23	2.1E-02
*Sphk1*	11	116543851	Exon (exon 1 of 9)	0.23	9.9E-05	0.24	2.5E-03

^a^ EWAS 1 tests for main effect of treatment with body weight, sex, and PCs as covariates; EWAS 2 same as EWASAS 1 and includes sex-by-treatment interaction.

### Relating to strain variation

Figure 1 Presents the mapping and analysis of the effects of CORT + DFP main effect and for CORT + DFP X sex interaction. Compared to control, CORT + DFP resulted in lower overall methylation and the sex by treatment interaction showed males to evince increased methylation and females to show decreased methylation overall.

**FIGURE 2:**
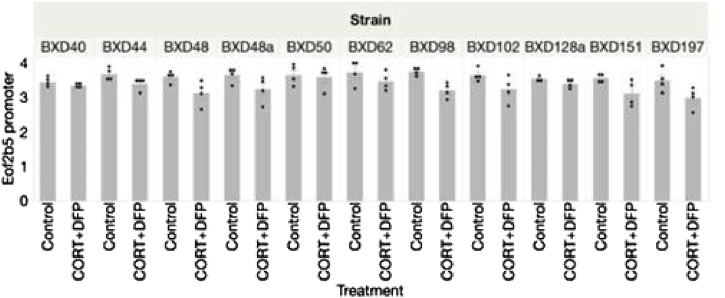
Strain distribution of methylation of *Eif2b5* 43 weeks following treatment with corticosterone and DFP.

The strain distribution of effect is presented in Figure 2. As illustrated in Figure 2, many of the CpGs show significant heterogeneity by genetic background, and the top hits from the EWAS shown in Figure 1a are the ones that are robust to such heterogeneity.

Analysis of the MBD-seq results showed significant associations between CORT + DFP for the following genes: *Eif2b5, Or2aj6, Nsun, Nlrp4e,* and *Cdh6*. The strongest signal was for *Eif2b5* (Figure 1a).

We then searched for genes that regulate *Eif2b5* using the mapping software available on Genenetwork.org. using the Genome-wide Efficient Mixed Model Association (GEMMA) method. GEMMA is software implementing Linear Mixed Model Association for genome-wide association studies (GWAS). Gene mapping in the BXD family of mice is analogous to GWAS in humans except for replication of genotypes. Several BXD mouse strains are closely related genetically, therefore the need to account for population stratification.

**FIGURE 3:**
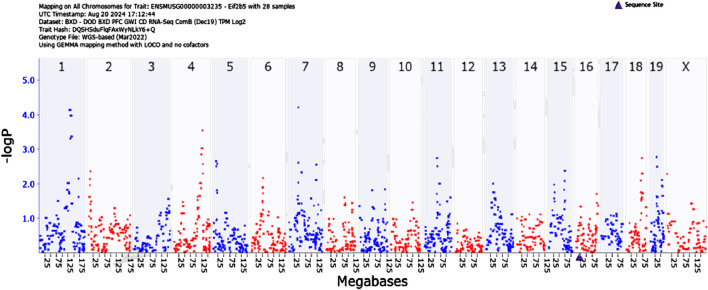
GEMMA quantitative trait analysis of *Eif2b5* gene expression. Note the significant association on Chromosome 1. Search for candidate genes in the interval reveals *Mapkapk2 (Mk2).*

Figure 3 illustrates quantitative trait loci analysis of *Eif2b5* expression. We observed significant associations between the expression phenotype and markers on chromosomes 1 and 7. The area under the QTL curve on chromosome 7 is in a gene-impoverished region; however, the QTL curve on chromosome 1 contains several genes among which is *Mapkapk2 (Mk2).*

**FIGURE 4:**
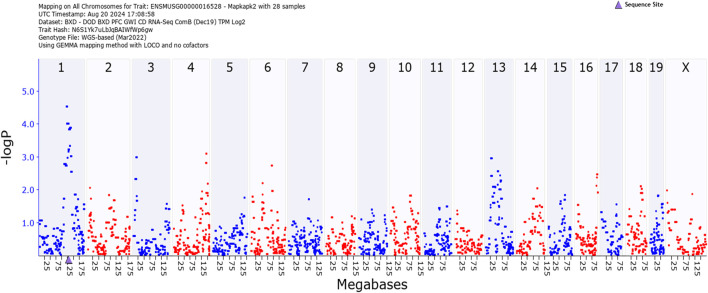
GEMMA quantitative trait analysis of *Mapkapk2* gene expression. Note the significant association on Chromosome 1. The peak is near the coding region (blue triangle on the X-axis). This indicates that the gene is *cis-*regulated.

Figure 4 illustrates quantitative trait loci analysis of *Mapkapk2* expression in hippocampus. The peak, indicating high association between expression and a marker near the coding region (blue triangle on the X-axis) shows that this gene is *cis*-regulated.

### What about corticosterone?

**FIGURE 5:**
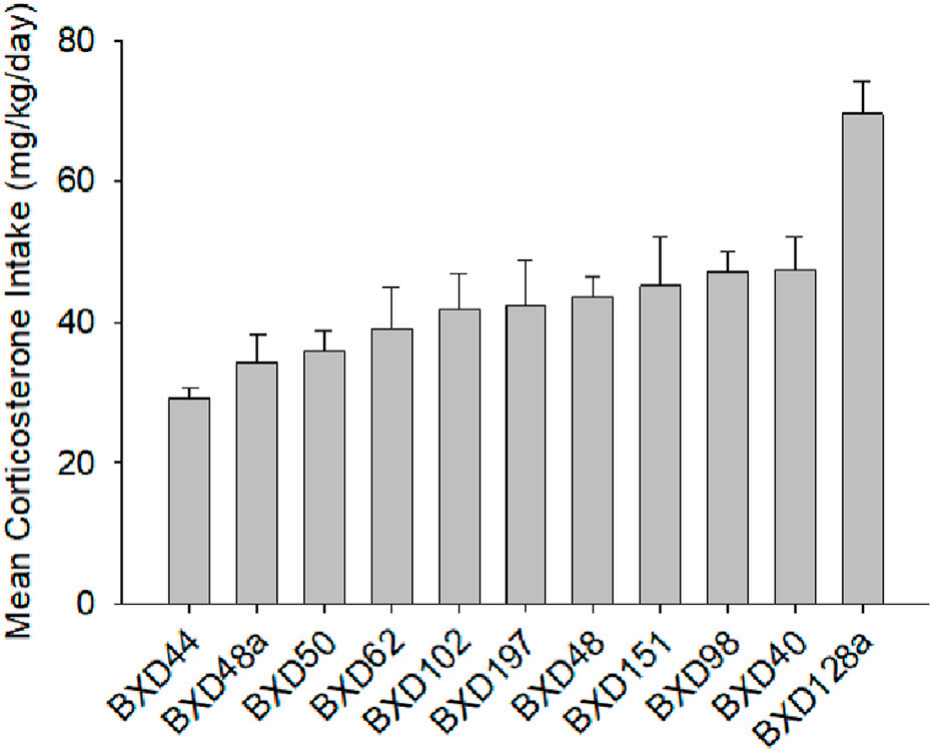
Mean daily consumption of corticosterone, males and females combined. Corticosterone intake represented here includes the pre DFP period and the weekly intakes for the 5 periods following DFP and the consumption on week 42. The data presented here are for both sexes the correlation for the sexes was r = 0.66, p < 0.01 one-tailed based on previous observation, so we combined the data for a single figure.

In our previous study on the genetic basis of susceptibility to develop GWI, we showed that corticosterone exacerbated the effect of DFP on the expression of *Il1b* (Jones et al., 2020). We also presented data showing strain (genetic) differences in corticosterone consumption. We then asserted that because analysis of covariance showed no influence of corticosterone intake, that differences in corticosterone ingestion had no effect on *Il1b* expression. Similarly, here we observed differences in corticosterone intake among the 11 strains (Figure 5).

The variation that we observed among the strains in corticosterone consumption did not correlate with differences in methylation of *Eif2b5.*

## Discussion

In this study, we focused on the hippocampus as those afflicted with GWI reported difficulties with memory (Li et al., 2011; Chao, 2023) and dysfunction of blood flow in this structure has been reported to be impaired with impairment increasing with age (Li et al., 2011). Also, we extend the findings of our previous small study involving just the parental strains of the BXD group and for 84 instead of 300 days (Mozhui et al., 2023). The small study was for proof of concept, and we identified four genes that had been differentially methylated by strain. The genes are *Ttll7,* which regulates glutamate as a neurotransmitter, *Akr1c14,* associated with the immune microenvironment, *Slc44a4,* a high-affinity, sodium-dependent choline transporter found in peripheral tissues and a choline supplier to other cells for membrane maintenance. *Rusc2* is associated with cognition. The present study expanded the number of BXD strains to 11 and extended the time from exposure to DFP to 300 days. The time frame approximates the developmental age of Gulf War veterans who are now in their early to late 50s. The current study expands the number of methylated candidate genes, including *Eif2b5, Or2aj6, Nsun, Nlrp4e, Cdh6. Eif2b5* is associated with myelination and GWI includes neurological problems involving deficits in myelin. *Or2aj6* participates in olfactory processing. Some Gulf War Veterans report loss of olfaction and this is often a prodromal sign of neurodegeneration (Chao, 2024), *Nsun2* is a methyltransferase and is associated with Dubowitz syndrome, an autosomal recessive disease that produces intellectual delay and microcephaly. At present there is no known association between GWI symptoms and this gene, *Nlrp4e* regulates actin cap formation during oögenesis and interferon 1 via inhibition of tank binding kinase 1. It has function in viral immunity and programmed cell death. Its involvement in GWI is presently unknown but seems to be a candidate for further study because of its role in neuroinflammation; *Cdh6,* cahedrin 6, is associated with gliomas. Its expression is positively associated with malignancy and negatively associated with prognosis. This is an important player in GWI as those so afflicted have increased risk for glioma. Of particular interest is our finding that *Eif2b5* is regulated by *Mapkapk2,* a gene that participates in cellular stress response, including production of the proinflammatory cytokines, *Tnfa, l1b,* and *IL6,* among several other cellular processes. The sex X treatment interaction for E*if2b5* was not unexpected. Previously, we showed a sex by treatment interaction for the acute effects of CORT + DFP (Jones et al., 2020) with female mice showing less neuroinflammation indices than males. In humans, the reverse has been observed with females showing more severe symptoms of GWI than their male counterpoints (Heboyan et al., 2019). As many of those afflicted with GWI are still experiencing debilitating symptoms are aging, the emphasis needs to be placed on treatments. Indeed, relatively few of those Gulf War veterans afflicted have shown recovery (Van Riper et al., 2017). One of the primary symptoms of chronic GWI is peripheral pain, originating from compromised central nervous system myelin (Abbink et al., 2019). Vanishing white matter (VWM) disease is a fatal disorder but apparently not related to the white matter compromise seen in GWI. This disease, triggered by stress, is seen primarily in children (Wong et al., 2019). A recent study [31] reported that a small molecule compound, 2BAct, reversed the loss of myelin in a mouse model of VWM. This compound stimulates *Eif2b5* expression. This or similar compounds may have promise for treating the chronic debilitating symptoms of GWI.

The value of our approach to show the genetic basis for individual differences in susceptibility to the acute and chronic manifestations of Gulf War Illness, shows the way to understanding the basic mechanisms of the diseases *per se.* Accordingly, this approach will point to the means for treatment or even prevention of the disease.

## Conclusion

Loss of myelin with associated musculoskeletal pain is a major and debilitating symptom of chronic GWI. It is likely that the loss of myelin relates to cognitive and other neurological symptoms as well. Here, we have shown a promising gene-based and biochemical mechanism that underlies chronic symptoms of GWI and the possibility of treating these symptoms. Because the GWI population is aging, time is the important factor in prosecuting the discovery of therapies.

## Data Availability

The original contributions presented in the study are publicly available. These data can be found here: https://info.genenetwork.org/infofile/source.php?GN_AccesionId=1063.
